# Morphological evolution indicates the transformation of stress interference in parallel fractures

**DOI:** 10.1038/s41598-025-32296-0

**Published:** 2026-01-08

**Authors:** Qianlong Zhou, Xiaodong Hu, Shaobo Han, Shou Ma, Fujian Zhou, Enjia Dong, Shu Jing

**Affiliations:** 1 College of Artificial Intelligence, China University of Petroleum, Beijing, Beijing, 102249 Beijing China; 2 Department of Mathematics and Physics, Leibniz University Hannover, Hannover, 30167 Hannover Germany; 3 SinoFTS Petroleum Services Ltd., Sinopec Huadong Oilfield Service Corporation, Beijing, 100101 Beijing China

**Keywords:** Hydraulic fracturing, Stress interference, Dual-fracture, Self-similarity, Quantitative characterization, Engineering, Solid Earth sciences

## Abstract

Fracture propagation is ubiquitous in natural strata and geological engineering, influencing key processes such as engineering stability assessment and design optimization. While prior studies have investigated the propagation of individual fractures through gelatin experiments, stress interference between fractures has been less considered. This study presents an innovative mirror-symmetric dual-fracture experiment that dynamically correlates fracture morphology with stress interference. Using light attenuation, we quantified the morphological evolution and identified distinct propagation regions governed by different degrees of stress interference. The results indicate that a regional transition occurs when the fracture radius approaches the fracture spacing. We defined a stress interference factor ($\beta$) to quantitatively characterize the interaction and derived an analytical model for $\beta$, which shows satisfactory agreement with experimental data. Through energy balance analysis during stagnation events, we revealed that the partitioning between elastic energy storage and viscous dissipation varies with fracture radius, explaining the observed differences in stagnation frequency and duration at different propagation stages.

## Introduction

Fracture propagation is a fundamental process in nature and engineering systems, critical to Carbon dioxide storage^1^, Magma migration^2,3^, geological tectonic changes^4,5^, and energy recovery^6^. In real situations, fracture propagation involves multiple fractures interacting through stress fields. The stress interaction between fractures influences their growth and morphology, forming complex fracture networks^7–9^. This study is crucial for advancing our understanding of fracture behavior under multi-fracture interactions. For example, reservoir stimulation^10,11^, transportation of formation magma^12^, and groundwater contamination issues^13^ involve fracture interactions and propagation.

Experimentally capturing the rapid process of fracture growth is challenging^14,15^. However, the use of transparent, elastic materials like gelatin^16^, combined with photometric techniques^17^, has proven effective for visualizing and tracking fracture propagation. Recent experimental studies on fluid-driven penny-shaped fractures have focused on the evolution of fracture morphology primarily, while stress interference between fractures has been less considered^18–21^. Few studies have explored the impact of stress interference on fracture morphology and propagation dynamics. Consequently, a critical gap exists in experimentally linking the dynamic stress field to morphological evolution, which is essential for understanding and predicting fracture network patterns in real geological settings.

In this paper, we address this gap by introducing a novel mirror-symmetric dual-fracture experimental apparatus. By employing light attenuation, we dynamically tracked and correlated the evolving fracture morphology with the intensifying stress interference. Our experiments successfully captured the distinct transition in propagation behavior when the fracture radius approaches the spacing, a key finding that prior single-fracture studies could not reveal. This work contributes to advancing our knowledge of how fractures may promote or inhibit each other’s growth under complex stress conditions.

## Photometric hydraulic fracture experiment

### Experimental setup

**Fig. 1:**
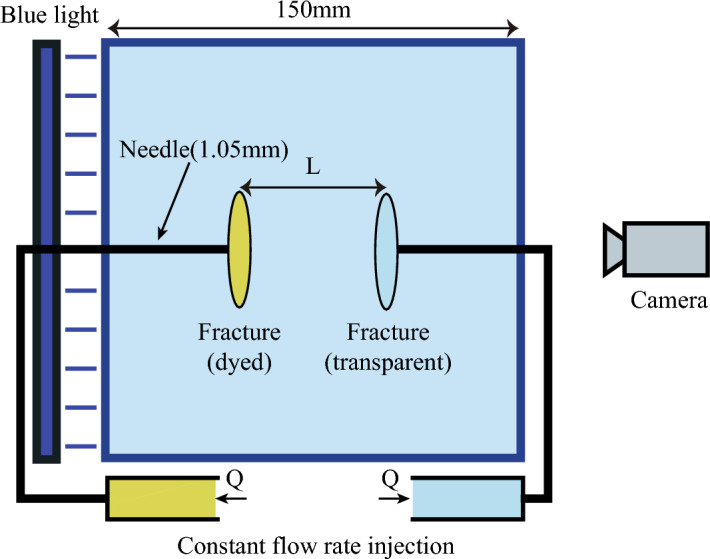
Schematic of the mirror-symmetric dual-fracture experiment experimental apparatus. We placed two injection needles opposite each other. Liquids were injected into the gelatin separately through two pumps. The liquid in one pump was dyed, while the other was transparent.

We developed a mirror-symmetric dual-fracture experimental apparatus to investigate stress interference effects on fracture propagation (Fig. 1). This system extends the single-fracture setup^19^ by incorporating two opposing injection needles aligned along a common axis. The distance *L* between needle tips defines the fracture spacing, selected to exceed fracture width during propagation. This design minimizes early-stage interference while ensuring directional control and avoiding boundary effects from the mold. Needles were embedded in a transparent gelatin mold (150 $\times$ 150 $\times$ 150 mm). A transparent circular Polyvinyl Chloride (PVC) sheet (5 mm in diameter, 0.5 mm in thickness) positioned at the needle tips fixed the radial propagation direction, guaranteeing parallel alignment of both fractures. To quantify fracture morphology, fluorescent dye was added to glycerin in one fracture. A uniform blue light source and cameras positioned on the opposite side enabled light attenuation measurements for tracking morphological evolution.

The Young’s modulus and Poisson’s ratio of the gelatin matrix in the experiment were controlled by adjusting the ratio to ensure the repeatability of the experiment. The Young’s modulus (*E*), Poisson’s ratio ($\nu$), and the viscosity ($\mu$) of the injected glycerol in the experiment were measured before the experiment. According to the parameters set up in the experiment, our experimental process is in the viscous-dominated regime. For the dual-fracture experiment, the disturbing stress increases gradually from zero. To verify the feasibility of the single-fracture model and to determine the error between the theoretical model and the experimental data, we first carried out the single-fracture crack propagation experiment before conducting the relatively symmetric fracture experiment. The control conditions between the single-fracture experiment and the dual-fracture experiment were consistent except for the set parameters.

The Young’s modulus and Poisson’s ratio of the gelatin matrix were controlled by adjusting its formulation, ensuring experimental reproducibility. Before experiments, we measured Young’s modulus, Poisson’s ratio (for varying gelatin formulations), and the viscosity of injected glycerol. All experiments commenced under viscosity-dominated conditions, consistent with the predefined parameters. In dual-fracture experiments, stress interference increased gradually from zero, with the entire process assumed to remain viscosity-dominated. To validate the single-fracture model and quantify discrepancies between theory and experiment, we performed single-fracture propagation tests before symmetric dual-fracture experiments. All experimental conditions (e.g., material properties, injection protocols) remained identical between single- and dual-fracture tests, except for the intentionally varied parameters.

### Materials and methods

Key experimental parameters included injection flow rate (*Q*), fluid viscosity ($\mu$), Young’s modulus of gelatin (*E*), and fracture spacing (*L*) (see Table 1 for symbol definitions). For dual-fracture experiments, *L*-defined as the distance between injection needles was set to 50 mm and 25 mm, while single-fracture tests had no defined spacing. All parameters (e.g., $\mu$, *E*) were measured before experiments and controlled to ensure reproducibility. Under these conditions, fracture propagation occurred in a viscosity-dominated regime (Appendix). The detailed experimental parameters are summarized in Table 2.

**Table 1:** Symbols and their definitions used in this paper.

**Symbol**	**Definition**
*L*	Fracture spacing
*Q*	Injection flow rate
*E*	Young’s modulus
$\nu$	Poisson’s ratio
$\mu$	Fluid viscosity
*t*	Time
*R*	Fracture radius
*W*	Fracture width (half-aperture)
$R_1$	Single-fracture radius
$R_2$	Dual-fracture radius
$\Delta R$	The difference between $R_1$ and $R_2$
$W_1$	Single-fracture width
$W_2$	Dual-fracture width
$\Delta W$	The difference between $W_1$ and $W_2$
$p_f$	Fluid pressure
$p_e$	Elastic stress
$p'$	Disturbing stress
$\beta$	The stress interference coefficient
*k*	Dimensionless prefactor from single-fracture scaling
$\alpha$	Time exponent from single-fracture scaling
$r_0$	Injection needle radius
$W_{\text {inj}}$	Injected energy
$W_{\text {ela}}$	Elastic energy stored in the gelatin matrix
$W_{\text {vis}}$	Viscously dissipated energy
$W_{\text {stick}}$	Width increment during stagnation
$t_{\text {stick}}$	Stagnation duration

**Table 2:** Parameter table for fracture experiment under fracture interference.

Experiment	Q (ml/min)	$\mu$ (Pa.s)	E (kPa)	L (mm)
exp1-1	10	1.0	380	-
exp1-2	10	2.0	380	-
exp1-3	5	2.0	380	-
exp1-4	10	0.5	380	-
exp1-5	10	2.0	570	-
exp2-1	10	1.0	380	25
exp2-2	5	1.0	380	25
exp2-3	10	2.0	570	25
exp2-4	10	1.0	570	25
exp2-5	10	1.0	380	50

### Experiment results

**Fig. 2:**
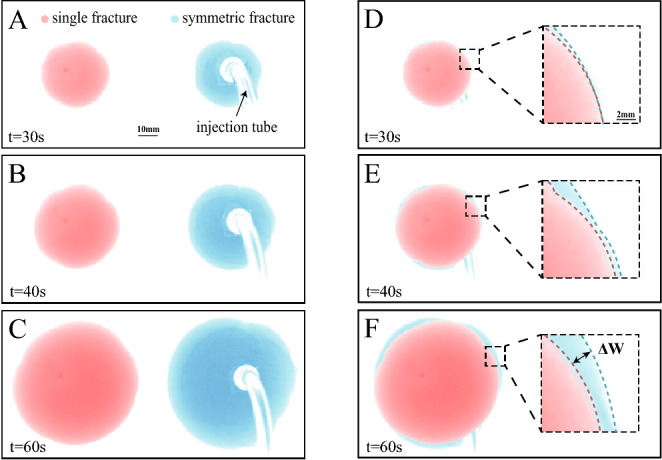
Morphological comparison of fracture propagation in single- and dual-fracture experiments. (**A**-**C**) Single-fracture results at t = 30, 40, 60 s; (**D**-**F**) Dual-fracture results at corresponding times. Red and blue curves represent single- and dual-fracture morphologies, respectively. Superimposed plots (A/D, B/E, C/F) highlight differences in fracture shapes. Note: Tubing artifacts are visible due to the injection tube.

**Fig. 3:**
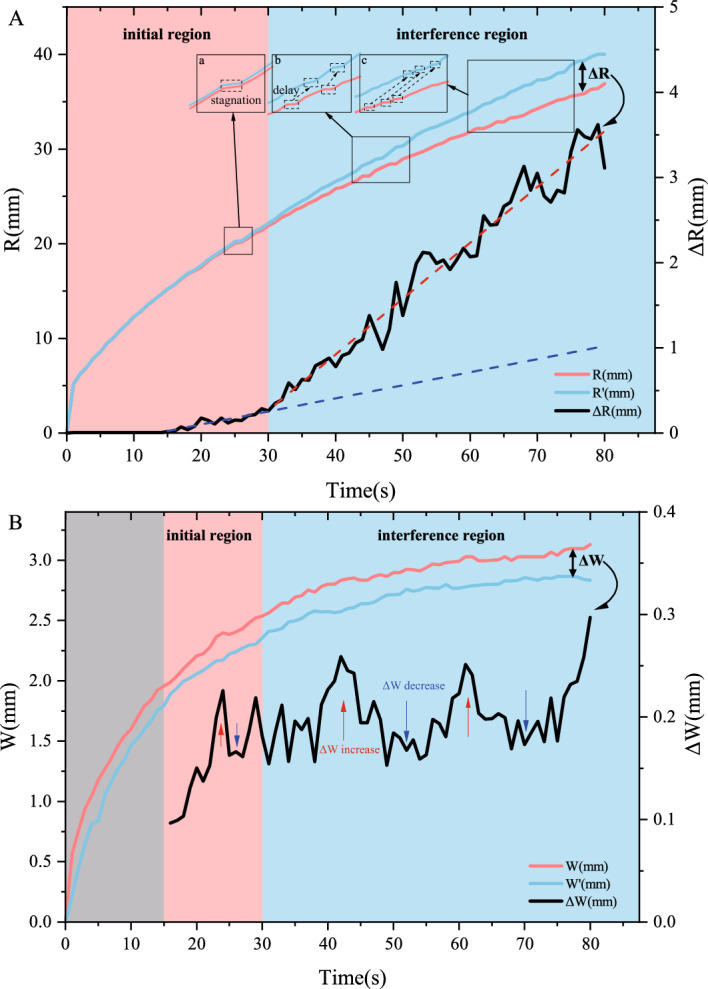
Temporal evolution of fracture dimensions in single-fracture experiments(exp1-1) and dual-fracture experiments(exp2-1). (**A**) Fracture radius comparison: Single-fracture (R1, red) and dual-fracture (R2, blue) radius over time. The radius difference ($\Delta R$, black) exhibits a marked transition near t = 30 s. Stagnation events (a, b, c) are observed as temporary halts in radius growth for both experiments. Blue and red dashed lines denote linear fits of $\Delta R$ during 15–30 s and post-30 s intervals, respectively. (**B**) Fracture width comparison: Single-fracture width (W1, red) and dual-fracture width (W1, blue) over time. The width difference ($\Delta W$, black) shows fluctuations corresponding to the stagnation events (Red/Black Arrow).

The morphological evolution of fractures under stress interference reveals distinct regimes, transitioning from an initial state of negligible interaction to a later stage dominated by fracture-to-fracture stress shadows. This transition is first visually apparent in the fracture shapes (Fig. 2) and is then quantitatively confirmed through the analysis of fracture dimensions over time (Fig. 3).

Fig. 2 presents a direct morphological comparison between single-fracture (red outlines) and dual-fracture (blue outlines) experiments at corresponding times. In the early stage ($t = 30$ s, Fig. 2A/D), the shapes of both fractures are nearly circular and overlap closely, indicating minimal interaction. As propagation continues ($t = 40$ s, Fig. 2B/E; $t = 60$ s, Fig. 2C/F), a clear divergence in morphology emerges. The dual fractures attain a larger area, indicating that the stress interference promotes radial expansion. (Note: The black arrows in Fig. 2A denote the location of the injection tube.)

In Fig. 3(A), we observe a marked increase in the radius difference ($\Delta R$) between single- and dual-fracture radius around $t=30$s. We define two regimes: the initial region ($t<30$s), which indicates stress interference is negligible, and the interference region ($t\ge 30$s), which indicates its effects dominate, and stress interference significantly alters fracture morphology.

Temporary stagnation in fracture radius growth occurs at $t=25$s, 48 s, and 65 s (labeled a, b, c in Fig. 3(A)). These stagnation events are consistent with stick-break instability reported in prior studies^22^-a phenomenon where fracture propagation proceeds through alternating stagnation (stick) and advancement (break) phases. In addition, we have observed the time delay of these stagnation events under stress interference. In Fig. 3(A)-a (initial region), the stagnation times for both single- and dual-fracture are nearly identical. However, in Fig. 3(A)-b and c (interference region), delays (dashed boxes) at the time points of radius stagnation are observed. Moreover, the delay in Fig. 3(A)-c is greater than that in Fig. 3(A)-b, indicating that stress interference intensifies this delay.

Notably, fluid-driven fractures propagate more slowly in gelatin compared to brittle materials like acrylic. Subtle stagnation events may be overlooked due to the 1-second interval of our image quantitative method. Fig. 3(B) presents the quantified width evolution, where early-stage measurements (before 15 s) exhibit significant variability due to injection tubing artifacts and pre-existing fractures. In the stable propagation stage, the single-fracture width ($W_1$) is greater than the dual-fracture width ($W_2$), and the width difference ($\Delta W$) exhibits different fluctuations in the two regions.

The fluctuations of $\Delta W$ are related to the delay of stagnation. When the radius stagnates and the fluid is injected constantly, causing a rapid increase in width. Fig. 3(A)-a,b,c presents a postponement of the stagnation events under a rising stress interference (the black arrow indicates a time delay of the stagnation events). The fluctuations of $\Delta W$ originate from asynchronous variations between $W_1$ and $W_2$, resulting from delays in stagnation events. Moreover, the prolongation of postponement time induces an extension in fluctuation duration.

## Mathematical model

Prior studies have characterized the morphological evolution of single fractures propagating under zero initial stress^19^. To investigate how stress interference alters propagation morphology, we first validated the rescaled dimensionless equations of single-fracture and got the dimensionless prefactor and exponent (Appendix).

For a single fracture propagating under zero confining pressure, the fluid pressure ($p_f$) equilibrates with the elastic stress ($p_e$) that sustains the fracture morphology. This equilibrium generates a stress field around the fracture. We assume the elastic stress acts normal to the fracture surface. In dual-fracture propagation, fractures experience stress interference from the opposing fracture. This interference arises from the normal stress field generated by the opposite fracture. During propagation, fractures are subjected to a disturbing stress ($p'$) induced by the opposite fracture’s growth^23^.

**Fig. 4:**
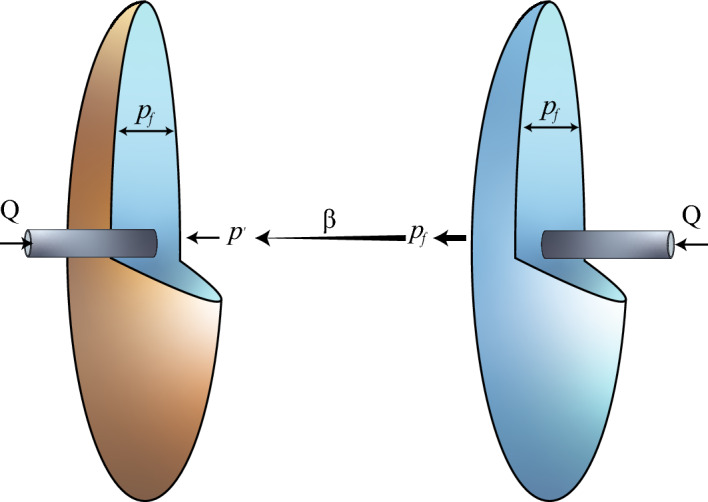
Stress interference relationship of mirror-symmetric fracture. The fluid pressures ($p_f$) in both fractures are identical. Taking the dyed fracture as the research subject, the fluid pressure in the transparent fracture is transmitted to the dyed fracture through $\beta$, generating a disturbing stress($p'$).

The relationship between stresses is derived from the assumptions:

1 $$\begin{aligned} p_f = p_e + p' \end{aligned}$$

Where $p_f$ is the fluid pressure within the fracture, $p_e$ is within the fracture, and $p'$ is the disturbed stress to which the fracture is subjected.

In our experiments, dual fractures propagate simultaneously under symmetric conditions. The magnitude and spatial effect of their stress fields are identical. Thus, the stress interference ($p'$) scales with fluid pressure ($p_f$) via a proportionality factor $\beta$:

2 $$\begin{aligned} p' = \beta \cdot p_f \end{aligned}$$

Building on the principles of volume conservation, lubrication theory, and elastic stress analysis under viscosity-dominated conditions, we extended the classical penny-shaped fracture propagation model to incorporate stress interference effects^19,24^.

3 $$\begin{aligned}&p_e \approx \frac{W}{R} \frac{E}{2(1-\sigma ^2)}, \end{aligned}$$

4 $$\begin{aligned}&\quad \frac{W}{t} \approx \frac{1}{3\mu } \frac{W^3 p_f}{R^2}, \end{aligned}$$

5 $$\begin{aligned}&\quad 4\pi WR^2 \approx Qt \end{aligned}$$

Based on the stress interference coefficient ($\beta$), we obtain the re-scaling relation of the fracture morphology under stress interference (e. g. radius):

6 $$\begin{aligned} R = \left( \frac{Q}{4\pi }\right) ^{1/3} \cdot (1-\beta )^{-1/9} \cdot \left( \frac{E}{6\mu (1-\nu ^2)}\right) ^{1/9} \cdot t^{4/9} \end{aligned}$$

This model is specifically formulated for a configuration of two parallel, radially propagating hydraulic fractures. The derivation is based on the theory of viscosity-dominated fracture propagation and assumes zero far-field stress or a uniform, isotropic in-situ stress field. This framework is designed to capture the stress interference effects observed in our experiments, which occur as the ratio of the fracture radius to spacing (R/L) evolves from much less than one to approximately one and beyond.

## Discussion

We get the rescaled data of single-fracture experimental measurements, which leads to a convincing collapse onto a single curve (Appendix). The best power law fit $R=k(Q/4\pi )^{1/3}\cdot (E/(6\mu \cdot (1-\nu ^2)))^{1/9}\cdot t^{\alpha }$ provide a dimensionless prefactor $k=0.435\pm 0.02$ and exponent $\alpha =0.50\pm 0.03$.

We consider the stress interference factor ($\beta$) dynamically quantifies the relationship between fluid pressure ($p_f$) and interference stress ($p'$). As fractures propagate, variations in the fracture stress field relative to fracture spacing (*L*) drive temporal changes in $\beta$, which is defined by Eq. (6):

7 $$\begin{aligned} \beta = 1 - R^{-9} \cdot k^9 \left( \frac{Q}{4\pi }\right) ^3 \cdot \left( \frac{E}{6\mu (1-\nu ^2)}\right) \cdot t^{9\alpha } \end{aligned}$$

**Fig. 5:**
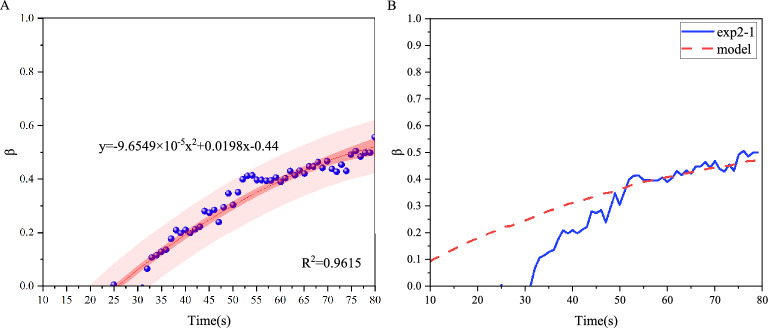
(**A**) Polynomial fitting of $\beta$(exp2-1) calculated by Eq. (7). (**B**) Comparison of experimental calculation results and models for $\beta$(exp2-1).

Assumes that the dynamic stress interference factor ($\beta$) inherently accounts for time-exponential variations induced by stress interactions. Consequently, the scaling prefactor (k) and time exponent ($\alpha$) derived from dual-fracture experiments align with those of single-fracture experiments. As shown in Fig. 5, the temporal evolution of $\beta$, calculated via Eq. (7), exhibits a nonlinear growth trend.

We observe that at $t=30$s, $\beta$ increases rapidly, which is consistent with the results of the stress regions we divided based on the fracture morphology changes. It is found that at this moment, the fracture radius is 22.16mm, and the fracture spacing between the two fractures is 25mm. Considering the intrinsic width of the two fractures, it can be considered that when the fracture radius is close to the fracture spacing, the interference stress between the fractures cannot be ignored.

For the interference region where the stress interference effect is obvious, we analyzed the $\beta$ change trend based on the stress interference model established by others. Following the established stress shadow theory^25–27^, we obtained the mathematical model of $\beta$. Through comparison with the experimental data, it is found that the experimental data are consistent with the model in the interference region (Fig. 5(B)).

8 $$\begin{aligned} \beta = 1 - \frac{L}{\sqrt{R^2 + L^2}} \end{aligned}$$

**Fig. 6:**
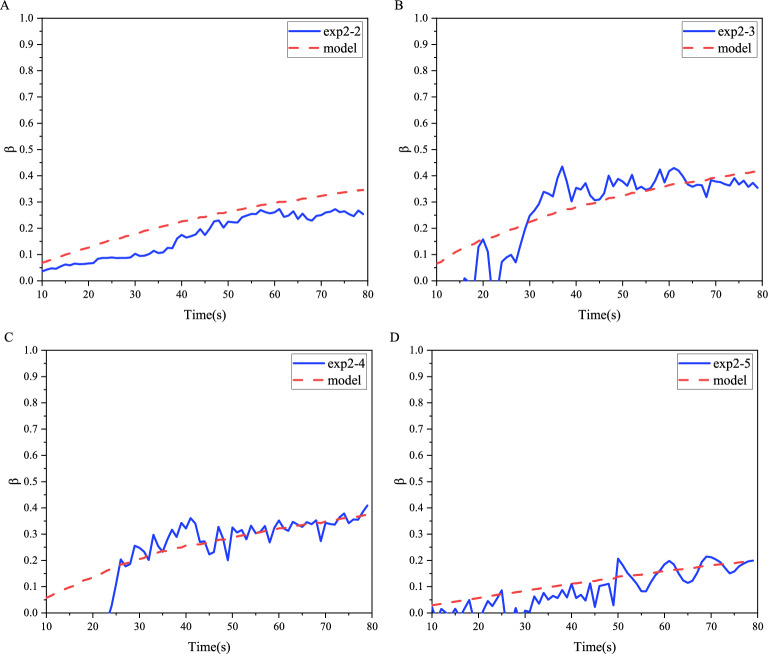
Comparison of experimental calculation results and models for $\beta$ under different experimental parameters.

We performed computational analyses on multiple sets of control experiments with varying parameters. Comparisons between calculated $\beta$ values and model predictions reveal obvious deviations during early-stage propagation. However, once the fracture radius approaches the fracture spacing ($R/L\approx 1$), experimental results and theoretical predictions converge with a fitting error of $18\%$ (Fig. 6), validating the model’s reliability.

Our experiments exhibited temporary stagnation in fracture propagation (stick-break instability) within gelatin matrices, which is possibly linked to a nucleation process that instigates break. During the break portion, energy dissipates primarily through viscous damping, while the stick portion represents energy accumulation at the fracture tip. The alternation of these portions reflects a requirement for sufficient energy re-accumulation or stress concentration recovery at the fracture tip to resume propagation.

During the stagnation stage of a single fracture, the system continues to inject fluid at a constant rate *Q*, and the fluid pressure equals the elastic stress ($p_f = p_e$). Although the fracture radius remains nearly constant, the fracture opening continues to grow. Neglecting surface energy, the energy balance can be written as^28,29^:

9 $$\begin{aligned} W_{\text {inj}} = W_{\text {ela}} + W_{\text {vis}}, \end{aligned}$$

where $W_{\text {inj}}$ denotes the injected energy, $W_{\text {ela}}$ is the elastic energy stored in the gelatin matrix, and $W_{\text {vis}}$ is the viscously dissipated energy. Using Eqs. (5)–(7), the individual energy terms are expressed as

10 $$\begin{aligned} W_{\text {inj}}&= Q \, p_f \, t, \end{aligned}$$

11 $$\begin{aligned} W_{\text {ela}}&= \frac{\pi }{2} \frac{p_e^2 R^3 (1-\nu ^2)}{E}, \end{aligned}$$

12 $$\begin{aligned} W_{\text {vis}}&= \frac{3\mu Q^2}{W^3} \ln \left( \frac{R}{r_0}\right) t, \end{aligned}$$

**Fig. 7:**
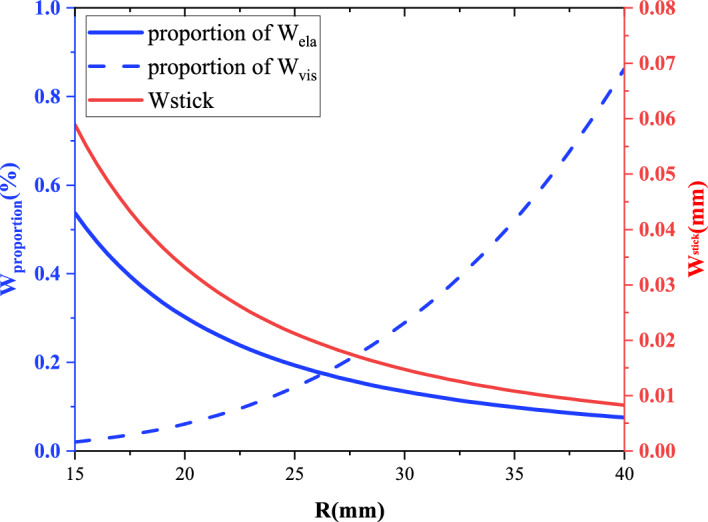
Changes in energy partitioning and fracture width during stagnation events at different radii.

The typical stagnation duration observed in Fig. 3A is $t_{\text {stick}} = 1~\text {s}$, and the associated width increment $W_{\text {stick}}$ at a given radius follows from Eq. (7). Evaluating Eqs. (10)–(12) at different radii during the stagnation period reveals how the injected energy partitions between elastic storage and viscous dissipation (Fig. 7).

Figure 7 illustrates that, at small radii, the stagnation-induced width increment $W_{\text {stick}}$ is relatively large, yielding a higher fraction of elastic energy. As the radius increases, $W_{\text {stick}}$ diminishes, elastic energy storage drops, and viscous dissipation rises sharply. This explains the observations in Fig. 3A: stagnation events occurring at small radii rapidly convert injected energy into elastic energy, leading to quick pressure build-up and fracture reactivation, so the stick phase is brief. At larger radii, the dominance of viscous dissipation prolongs and multiplies stagnation episodes, as evident when comparing Fig. 3A-a, b, and c.

As a soft viscoelastic material, gelatin exhibits prolonged stagnation time and slower propagation rates (1 s stagnation time in Fig. 3(A)-a,b,c), due to its viscosity-dominated energy dissipation mechanisms. Under stress interference, the stagnation stage exhibits delayed initiation (the black arrow in Fig. 3(A)-a,b,c) due to a significant increase in viscous dissipation.

## Conclusion

1. We developed a mirror-symmetric dual-fracture experimental method to correlate stress fields with fracture morphology evolution dynamically, and quantitatively analyzed fracture morphology under stress interference.
2. Through quantitative analysis of fracture morphology, we observed stress interference effects on fracture configurations and defined distinct stress interference zones based on morphological variations. Additionally, we identified more pronounced stick-break instability in elastic-brittle matrices during fracture propagation.
3. We defined an influence factor $\beta$ to characterize stress interaction effects, and its nonlinear growth (particularly as the fracture radius approaches spacing) demonstrates stage-dependent stress interference mechanisms.
4. We established a mathematical model to analyze stress interference effects on fracture morphology in a parallel, mirror-symmetric fracture system. The model is derived from governing equations for viscosity-dominated propagation and has been validated under corresponding experimental conditions. It effectively captures the critical transition in stress interference that occurs as the fracture radius-to-spacing ratio (R/L) approaches and surpasses unity.
5. Through energy balance analysis during stagnation events, we demonstrated that the partitioning between elastic energy storage and viscous dissipation varies significantly with fracture radius. At small radii, elastic energy storage dominates, leading to rapid pressure build-up and brief stagnation phases. As the radius increases, viscous dissipation becomes dominant, prolonging stagnation episodes.

## Supplementary Information

Below is the link to the electronic supplementary material.

Supplementary material 1.

## Data Availability

The data that support the findings of this study are available from the corresponding author (huxiaodong@cup.edu.cn) upon reasonable request.
