# Supplementary material for: Morphological evolution indicates the transformation of stress interference in parallel fractures
Source: Sci Rep. 2026 Jan 8;16:2520. doi: 10.1038/s41598-025-32296-0 (PMC12819401; doi:10.1038/s41598-025-32296-0)
Supplement: Supplementary file 1 — Supplementary material 1. [file 41598_2025_32296_MOESM1_ESM.pdf]

Appendix: Morphological evolution indicates the transformation of stress interference in parallel fractures

Qianlong Zhou[1], Xiaodong Hu[1*], Shaobo Han[2], Shou Ma[3], Fujian Zhou[1], Enjia Dong[1], Shu Jing[1]

[1*]College of Artificial Intelligence, China University of Petroleum, Beijing, Beijing, 102249, Beijing, China.
[2]Department of Mathematics and Physics, Leibniz University Hannover, Hannover, 30167, Hannover, Germany.
[3]SinoFTS Petroleum Services Ltd., Sinopec Huadong Oilfield Service Corporation, Beijing, 100101, Beijing, China.

*Corresponding author(s). E-mail(s): huxiaodong@cup.edu.cn; Contributing authors: 2024310816@student.cup.edu.cn; shaobohan@iop.uni-hannover.de; shou.ma@sinofts.com; zhoufj@cup.edu.cn; 1165309373@qq.com; 13167591023@163.com;

Appendix A The viscous-toughness criterion

In fluid-driven fracturing experiments, identifying whether energy dissipation is dominated by viscous flow resistance or by the energy required to create new fracture surfaces is essential for selecting the appropriate theoretical framework. To distinguish the viscous and toughness contributions during crack propagation, we define a dimensionless pressure ratio as follows:

$$\frac{\Delta p_f}{\Delta p_v} = \frac{\gamma_s^{1/2} E^{1/2} W^3}{\mu Q R^{1/2}}, \tag{A1}$$

where Δp_f and Δp_v represent the toughness-related and viscous pressure drops, respectively, γ_s is the surface energy, E is the Young's modulus, W is the fracture width, R is the fracture radius, Q is the injection rate, and μ is the fluid viscosity.

By substituting the characteristic scalings for toughness-dominated propagation into Eq. (A1), we obtain the corresponding toughness pressure ratio:

$$\left[\frac{\Delta p_f}{\Delta p_v}\right]_t = \frac{(1-\nu^2)^{13/5}\gamma_s^{9/5}t^{2/5}}{\mu Q^{3/5}E^{4/5}}, \tag{A2}$$

where ν denotes Poisson's ratio and t is time. The viscous pressure ratio relates to the toughness ratio through

$$\left[\frac{\Delta p_f}{\Delta p_v}\right]_v = \left(\left[\frac{\Delta p_f}{\Delta p_v}\right]_t\right)^{5/18}. \tag{A3}$$

A toughness-dominated regime is identified when $[\Delta p_f/\Delta p_v]_t \gg 1$, whereas a viscosity-dominated regime occurs when $[\Delta p_f/\Delta p_v]_v \gg 1$. Using the experimental parameters of this study, the computed viscous pressure ratio satisfies $[\Delta p_f/\Delta p_v]_v \gg 1$ throughout propagation, indicating that crack growth remains firmly within the viscosity-dominated regime.

Appendix B Governing equations

B.1 Single-fracture formulation

For a fluid-driven axisymmetric disc-shaped fracture of radius $R(t)$ and half-aperture $W(r,t)$ propagating under negligible initial stress and shear effects, the internal fluid pressure $p_f(r,t)$ equilibrates with the elastic stress $p_e(r,t)$ normal to the fracture faces. The governing relations combining elastic response, lubrication flow, and volume conservation in the viscous-dominated regime are[1, 2]

$$p_e \approx \frac{W}{R}\frac{E}{2(1-\nu^2)}, \tag{A4}$$

$$\frac{W}{t} \approx \frac{1}{3\mu}\frac{W^3 p_f}{R^2}, \tag{A5}$$

$$4\pi W R^2 \approx Qt. \tag{A6}$$

This model is specifically formulated for a configuration of two parallel, radially propagating hydraulic fractures. The derivation is based on the theory of viscosity-dominated fracture propagation and assumes zero far-field stress or a uniform, isotropic in-situ stress field. This framework is designed to capture the stress interference effects observed in our experiments, which occur as the ratio of the fracture radius to spacing (R/L) evolves from much less than one to approximately one and beyond.

Combining Eqs. (A4)–(A6) yields the self-similar single-fracture scalings for radius, thickness, and elastic stress:

$$R \approx \left(\frac{Q}{4\pi}\right)^{1/3}\left(\frac{E}{6\mu(1-\nu^2)}\right)^{1/9} t^{4/9}, \tag{A7}$$

$$W \approx \left(\frac{Q}{4\pi}\right)^{1/3}\left(\frac{E}{6\mu(1-\nu^2)}\right)^{-2/9} t^{1/9}, \tag{A8}$$

$$p_e \approx \frac{E}{2(1-\nu^2)} \left(\frac{E}{6\mu(1-\nu^2)} \right)^{-1/3} t^{-1/3}. \tag{A9}$$

B.2 Dual-fracture formulation

For mirror-symmetric dual fractures, the internal fluid pressure of each crack must overcome both the local elastic stress and the disturbing stress transmitted from the opposing fracture. The stress equilibrium is

$$p_f = p_e + p', \tag{A10}$$

where the disturbing stress is assumed proportional to the fluid pressure through the stress interference coefficient β:

$$p' = \beta\, p_f. \tag{A11}$$

Substituting Eq. (A11) into Eq. (A10) gives

$$p_f = p_e + \beta p_f. \tag{A12}$$

Applying the same elastic–hydrodynamic framework as the single-fracture case while accounting for the reduction of effective driving stress by $(1-\beta)$ leads to the dual-fracture scalings:

$$R = \left(\frac{Q}{4\pi}\right)^{1/3} (1-\beta)^{-1/9} \left(\frac{E}{6\mu(1-\nu^2)}\right)^{1/9} t^{4/9}, \tag{A13}$$

$$W = \left(\frac{Q}{4\pi}\right)^{1/3} (1-\beta)^{2/9} \left(\frac{E}{6\mu(1-\nu^2)}\right)^{-2/9} t^{1/9}, \tag{A14}$$

$$p_e = \frac{E}{2(1-\nu^2)} (1-\beta)^{1/3} \left(\frac{E}{6\mu(1-\nu^2)}\right)^{-1/3} t^{-1/3}. \tag{A15}$$

B.3 Stress interference coefficient

Fluid pressure propagating through the elastic matrix generates stress interactions that decay with fracture spacing L. Following the stress transmission solutions for penny-shaped fractures[3–6], the disturbing stress at a distance L from the source fracture is

$$p' = p_f \left[1 - \frac{\overline{L}}{\sqrt{\overline{L}_1 \overline{L}_2}} \cos\left(\theta - \frac{\theta_1+\theta_2}{2}\right) - \frac{\overline{L}}{(\overline{L}_1 \overline{L}_2)^{3/2}} \sin\theta \sin\left(\frac{3}{2}(\theta_1+\theta_2)\right) \right], \tag{A16}$$

where barred quantities denote normalized distances and θ represents the angular position relative to the fracture plane. Under the symmetric configuration and orientation of our experiments, Eq. (A16) simplifies to a compact expression for β:

$$\beta = 1 - \frac{L}{\sqrt{R^2 + L^2}}. \tag{A17}$$

This relation captures the transition from weak interference ($R \ll L$, $\beta \to 0$) to strong interference ($R \approx L$, $\beta \to 1$) observed in our measurements.

Appendix C Experimental results fit

According to experimental measurements under different parameters, we rescale the raw data (Fig. A1) of single fracture for R, we get the dimensionless prefactor of $k = 0.435 \pm 0.02$ and exponent $\alpha = 0.50 \pm 0.03$.

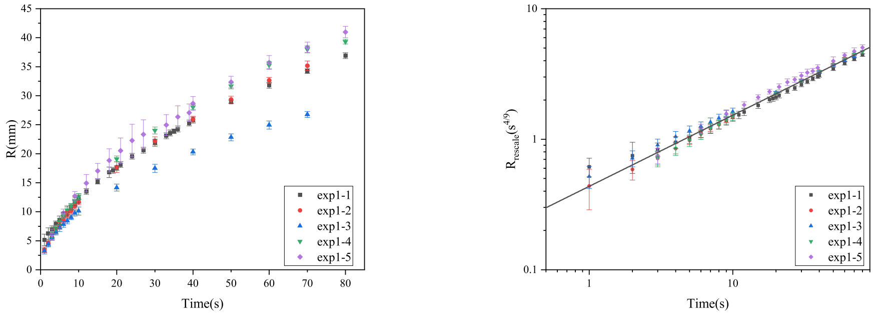

Fig. A1 Raw data (right) and rescaled data (left) of single-fracture experiments.

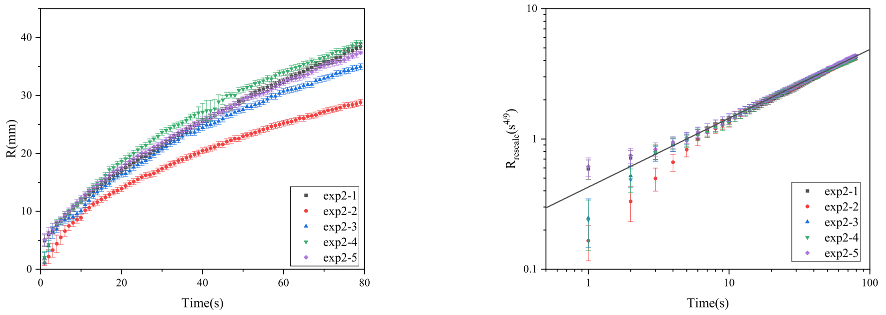

Fig. A2 Raw data (right) and rescaled data (left) of dual-fracture experiments.

Based on the stress interference coefficient (β), we obtain the re-scaling relation of the fracture morphology under stress interference (e. g. radius):

$$R = \left(\frac{Q}{4\pi}\right)^{1/3} \cdot (1-\beta)^{-1/9} \cdot \left(\frac{E}{6\mu(1-\nu^2)}\right)^{1/9} \cdot t^{4/9} \tag{A18}$$

Next, we show the raw data (Fig. A2) from the dual fracture experiment, as well as rescaled data based on the single fracture power law fit results and scaling Eq. (A18), which leads to a convincing collapse onto a single curve in Fig. A2.

Appendix D Experimental details

In our experiment, gelatin matrices with different particle concentrations were prepared to obtain a range of elastic properties. Table A1 lists the detailed concentration, solidification time, curing temperature, and measured Young's modulus for each formulation.

Table A1 Parameters of gelatin matrix configuration (Table A1).

Concentration	Solidification time (h)	Temperature (°C)	Young's modulus (kPa)
4%	24	16	140
8%	24	16	380
12%	24	16	570

Here, we present the photometric image used for quantifying fracture morphology and its side-view image of exp 2-1 (t = 35 s). In the photometric image, dark regions represent the dyed fracture, while the transparent fracture is discernible solely along its edges. For the side-view image, light propagates from left to right (Fig. A3).

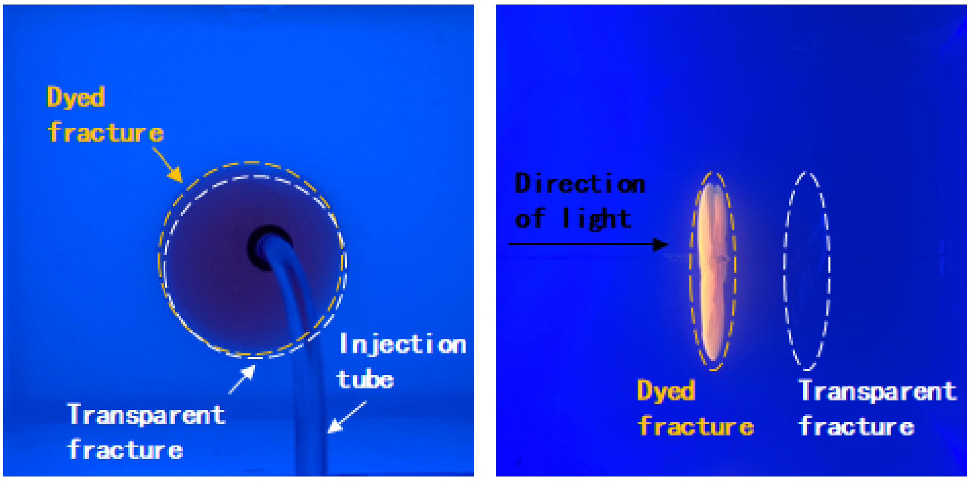

Fig. A3 Light attenuation picture and side view of t=35s.

References

[1] Lai, C.-Y., Zheng, Z., Dressaire, E., Wexler, J.S., Stone, H.A.: Experimental study on penny-shaped fluid-driven cracks in an elastic matrix. Proc. R. Soc. A **471**(2182) (2015) https://doi.org/10.1098/rspa.2015.0255

[2] Savitski, A.A., Detournay, E.: Propagation of a penny-shaped fluid-driven fracture in an impermeable rock: asymptotic solutions. Int. J. Solids Struct. **39**(26), 6311–6337 (2002) https://doi.org/10.1016/s0020-7683(02)00492-4

[3] Itou, S.: Effect of a penny-shaped interface crack on elastic wave propagation. Int. J. Solids Struct. **49**, 1065–1072 (2012) https://doi.org/10.1016/j.ijsolstr.2012.01.007

[4] Sneddon, I.N.: The distribution of stress in the neighborhood of a crack in an elastic solid. Proc. R. Soc. A **187**, 299–260 (1946) https://doi.org/10.1098/rspa.1946.0077

[5] Spence, D.A., Sharp, P.: Self-similar solutions for elastohydrodynamic cavity flow. Proc. R. Soc. A **400**, 289–313 (1985) https://doi.org/10.1098/rspa.1985.0081

[6] Wu, R., Kresse, O., Weng, X., Cohen, C., Gu, H.: Modeling of interaction of hydraulic fractures in complex fracture networks (2012) https://doi.org/10.2118/152052-MS
